# Prion Seeding Activities of Mouse Scrapie Strains with Divergent PrP^Sc^ Protease Sensitivities and Amyloid Plaque Content Using RT-QuIC and eQuIC

**DOI:** 10.1371/journal.pone.0048969

**Published:** 2012-11-05

**Authors:** Sarah Vascellari, Christina D. Orrù, Andrew G. Hughson, Declan King, Rona Barron, Jason M. Wilham, Gerald S. Baron, Brent Race, Alessandra Pani, Byron Caughey

**Affiliations:** 1 Laboratory of Persistent Viral Diseases, Rocky Mountain Laboratories, National Institute of Allergy and Infectious Diseases, National Institutes of Health, Hamilton, Montana, United States of America; 2 Department of Biomedical Sciences, University of Cagliari, Monserrato, Italy; 3 Division of Neurobiology, The Roslin Institute and R(D)SVS, University of Edinburgh, Roslin, Midlothian, United Kingdom; Creighton University, United States of America

## Abstract

Different transmissible spongiform encephalopathy (TSE)-associated forms of prion protein (e.g. PrP^Sc^) can vary markedly in ultrastructure and biochemical characteristics, but each is propagated in the host. PrP^Sc^ propagation involves conversion from its normal isoform, PrP^C^, by a seeded or templated polymerization mechanism. Such a mechanism is also the basis of the RT-QuIC and eQuIC prion assays which use recombinant PrP (rPrP^Sen^) as a substrate. These ultrasensitive detection assays have been developed for TSE prions of several host species and sample tissues, but not for murine models which are central to TSE pathogenesis research. Here we have adapted RT-QuIC and eQuIC to various murine prions and evaluated how seeding activity depends on glycophosphatidylinositol (GPI) anchoring and the abundance of amyloid plaques and protease-resistant PrP^Sc^ (PrP^Res^). Scrapie brain dilutions up to 10^−8^ and 10^−13^ were detected by RT-QuIC and eQuIC, respectively. Comparisons of scrapie-affected wild-type mice and transgenic mice expressing GPI anchorless PrP showed that, although similar concentrations of seeding activity accumulated in brain, the heavily amyloid-laden anchorless mouse tissue seeded more rapid reactions. Next we compared seeding activities in the brains of mice with similar infectivity titers, but widely divergent PrP^Res^ levels. For this purpose we compared the 263K and 139A scrapie strains in transgenic mice expressing P101L PrP^C^. Although the brains of 263K-affected mice had little immunoblot-detectable PrP^Res^, RT-QuIC indicated that seeding activity was comparable to that associated with a high-PrP^Res^ strain, 139A. Thus, in this comparison, RT-QuIC seeding activity correlated more closely with infectivity than with PrP^Res^ levels. We also found that eQuIC, which incorporates a PrP^Sc^ immunoprecipitation step, detected seeding activity in plasma from wild-type and anchorless PrP transgenic mice inoculated with 22L, 79A and/or RML scrapie strains. Overall, we conclude that these new mouse-adapted prion seeding assays detect diverse types of PrP^Sc^.

## Introduction

Misfolding of cellular prion protein (PrP^C^) into the scrapie prion protein (PrP^Sc^) isoform is a key event in the pathogenesis of prion disorders [1], [2]. PrP^Sc^ is the main component of the TSE infectious agent [3]–[8] and is able to propagate itself by seeding and templating a conformational change in PrP^C^, a glycosylphosphatidylinositol (GPI)-anchored glycoprotein [2], [4], [9], [10]. Unlike PrP^C^, PrP^Sc^ tends to be aggregated [11]–[15], partially resistant to proteases [3], [14], rich in beta sheet [16]–[20], and lacking in native alpha helices [16], [18], [19].

In the brain PrP^Sc^ can accumulate in deposits ranging from large fibrillar amyloid plaques [21]–[24] to smaller diffuse non-amyloid oligomers [25], [26]. Diffuse forms are predominant in many human and animal TSEs. However, PrP^Sc^ amyloid is a prominent feature of some genetic human prion diseases such as Gerstmann-Sträussler-Scheinker syndrome (GSS) [27] and prion protein cerebral amyloid angiopathy (PrP-CAA) [28]. In numerous TSE types, both amyloid and non-amyloid deposits can be found in the same tissue. However, in scrapie-infected transgenic mice expressing prion protein lacking the glycosylphosphatidylinositol anchor (GPI), PrP^Sc^ appears to be exclusively contained in amyloid plaques [29], [30]. Both large amyloid fibrils and non-amyloid aggregates of PrP^Sc^ are associated with high levels of infectivity [13], [29], but smaller non-fibrillar oligomers have been found to have the highest specific infectivity per unit protein with several scrapie strains [13], [31]. Nonetheless, the relative contributions of different PrP^Sc^ aggregates to prion propagation and TSE pathogenesis in vivo remains unclear.

Protease-resistant PrP^Sc^ (PrP^Res^) is often used as a definitive biological marker for TSE infections, but several studies have shown that infectivity is not always well-correlated with PrP^Res^ level [32]–[35]. Indeed, infectivity can sometimes be associated with forms of PrP^Sc^ that are largely proteinase K (PK)-sensitive (sPrP^Sc^) [36]. For instance, when inoculated into knock-in transgenic mice homozygous for P101L PrP^C^ (101LL), 139A scrapie leads to high PrP^Res^ [37] and infectivity levels in the brain while 263K scrapie elicits similarly high infectivity levels but little or no PrP^Res^ [35]. These and many other observations emphasize the diversity of abnormal TSE-associated PrP structures.

The ability to detect various types of PrP^Sc^ is important in TSE diagnostics. A number of cell-free reactions have emerged which allow highly sensitive PrP^Sc^ detection based on in vitro prion-seeded polymerization and conformational conversion of brain-derived PrP^C^ or recombinant PrP^C^ (rPrP^Sen^) (for reviews, see [38], [39]). Among the most sensitive, rapid and practical of these assays are the real time quaking induced conversion (RT-QuIC) [40]–[43] and enhanced QuIC (eQuIC) [44] assays. RT-QuIC is a shaken, multi-well plate-format reaction that is based on the detection of PrP^Sc^-seeded recombinant PrP amyloid fibrils using an amyloid-sensitive fluorescent dye, thioflavin T (ThT). In an end-point dilution mode, RT-QuIC can be quantitative in a manner that is conceptually analogous to the end-point dilution titrations classically used in animal bioassays [41], [45]. The eQuIC assay incorporates the use of a selective conformational antibody 15B3 to capture PrP aggregates in biological fluids such as blood plasma [44]. However, the extent to which divergent types of PrP^Sc^ can seed the polymerization of PrP^C^ into amyloid fibrils is not clear.

Building on recent successes in using the RT-QuIC and eQuIC reactions to amplify small amounts of hamster, sheep, cervid and human PrP^Res^ [39], [41]–[47], we have now adapted these assays to murine-adapted scrapie strains to explore how prion seeding activity in these assays depends on PrP^Sc^ i) GPI anchoring, ii) amyloid vs non-amyloid ultrastructure, and iii) PK-sensitivity. Moreover, the availability of mouse TSE-adapted RT-QuIC and eQuIC reactions should facilitate fundamental studies of TSE diseases because mouse models are used extensively to reveal the biological principles of prion transmission and pathogenesis.

## Results

### Development of a mouse RT-QuIC assay

**Figure 1:**
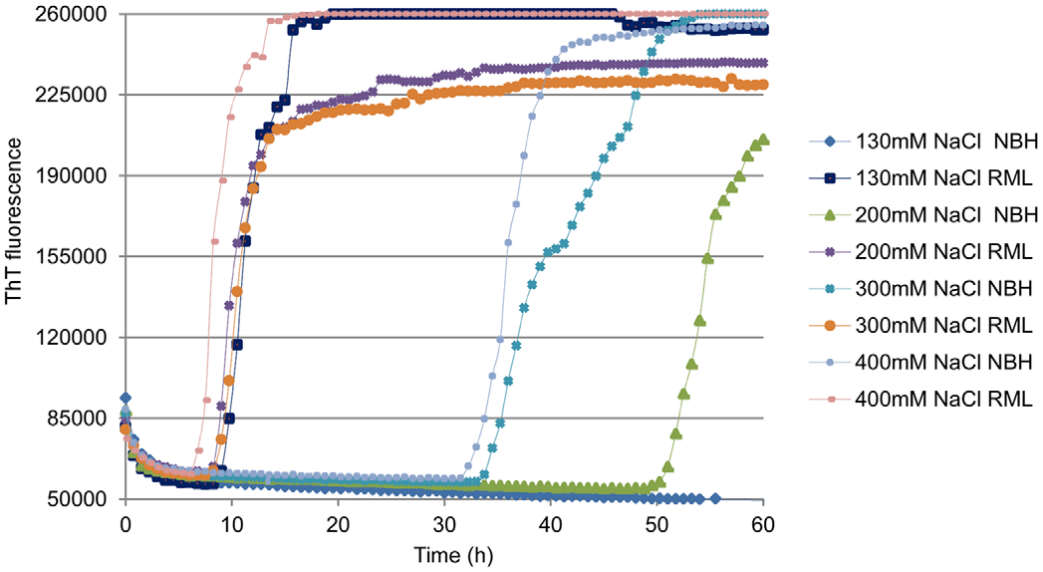
RT-QuIC NaCl titration in mouse RML scrapie model using moPrP^C^23–231 as substrate. RT-QuIC reactions were seeded with 5×10^−6^ dilutions of normal brain homogenates (NBH) and WT RML scrapie BH containing ∼200 fg of PrP^Res^. Final concentrations of 130, 200, 300 and 400 mM NaCl were used. The vertical axis indicates the average ThT fluorescence from four replicate wells.

Previous studies have indicated that two key interactive parameters in the development of RT-QuIC reactions for new prion strains and host species are the rPrP^Sen^ substrate and the NaCl concentration in the RT-QuIC buffer [41], [43], [44]. To adapt the RT-QuIC reaction to the detection of mouse PrP^Sc^, we tested different NaCl concentrations in combination with either full-length mouse rPrP^Sen^ residues 23–231 (moPrP^Sen^23–231) or N-terminally truncated mouse rPrP^Sen^ residues 90–231 (moPrP^Sen^ 90–231) as substrates. Using 130 mM NaCl in combination with moPrP^Sen^ 23–231, we could detect 5×10^−6^ brain tissue dilutions containing ∼200 fg of PrP^Res^ from RML scrapie-infected wild-type (WT) mice. No spontaneous (unseeded) fibrillization of rPrP^Res^ amyloid (rPrP^spon^) was detected in control reactions containing normal brain homogenate (NBH) (Figure 1). In contrast, NBH controls gave rPrP^spon^ when moPrP^Sen^23–231 was used with higher NaCl concentrations (≥200 mM). When using moPrP^C^ 90–231 as substrate, rPrP^spon^ generation was observed within 20–30 h with all NaCl concentrations tested (130–400 mM; data not shown).

### RT-QuIC of additional mouse-adapted scrapie strains in wild-type mice

**Figure 2:**
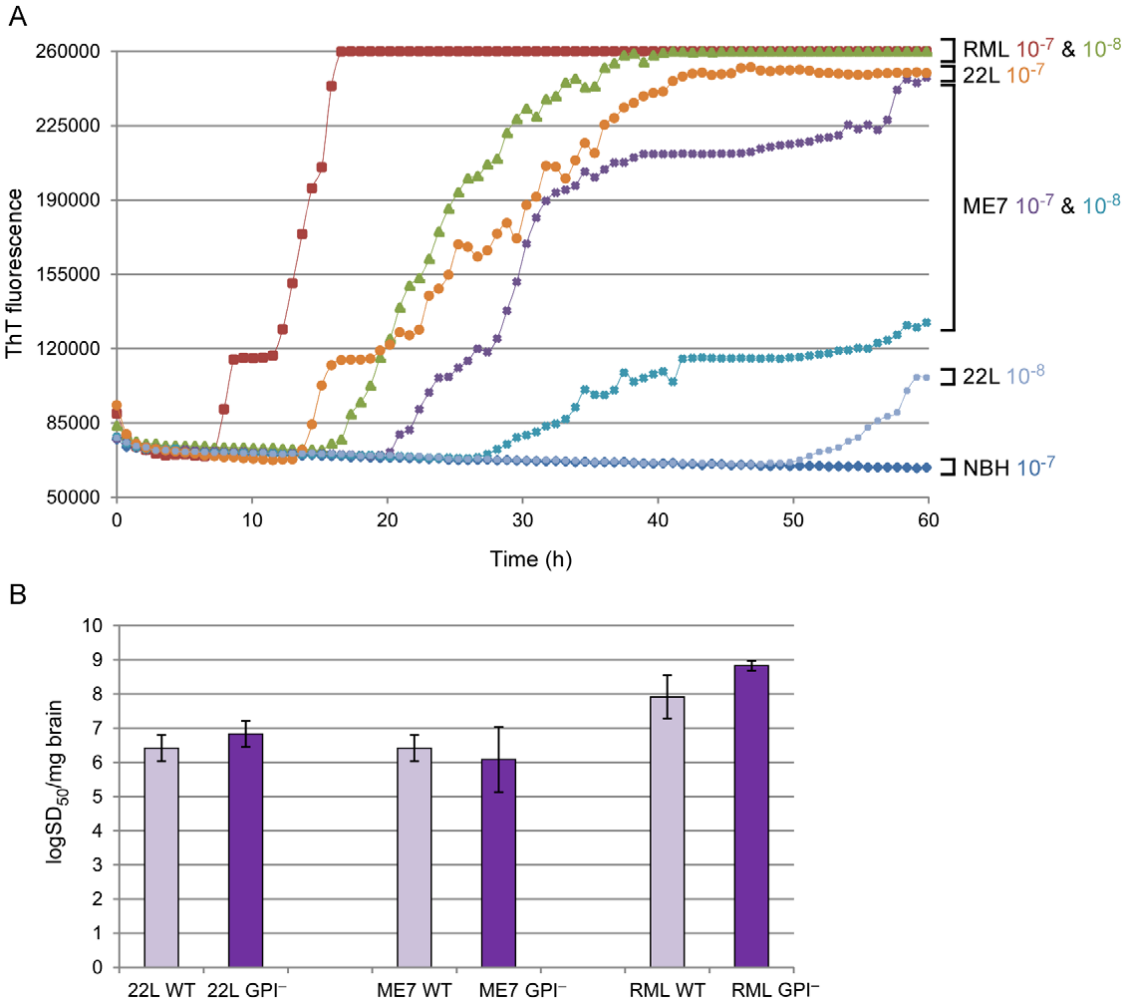
RT-QuIC comparison of multiple mouse-adapted scrapie strains. (**A**), Brain tissues dilutions (5×10^−7^ and 5×10^−8^) from WT mice infected with 22L, ME7 and RML scrapie strains were used to seed RT-QuIC reactions containing moPrP^Sen^23–231 substrate. A final concentration of 130 mM NaCl was used for the reaction. The average ThT fluorescence from a set of quadruplicate wells is reported on the vertical axis. (**B**), RT-QuIC end-point dilution analysis of brain homogenates from WT (light purple bars) and GPI^–^ (dark purple bars) mice infected with 22L, ME7 and RML. Four replicate wells were used for each brain homogenates dilution. The means ±SD of Spearman-Kärber estimates of the SD_50_/mg brain tissue from three different experiments are shown.

To gauge the strain-dependence of murine RT-QuIC analyses, we also analyzed the 22L and ME7 scrapie strains in brain homogenates from clinically affected WT C57BL/10 mice (Figure 2A). With both of these strains, seeding activity was detected in all replicate reactions seeded with dilutions of 5×10^−7^. Such dilutions contained ∼20 fg PrP^Res^ as estimated by semiquantitative immunoblotting of PK-treated brain homogenates (data not shown). The rapid negative-to-positive conversion of individual wells occasionally caused the stepwise increases in the fluorescence averaged from all wells. With the RML strain, uniformly positive replicates were obtained with 5×10^−8^ dilution containing ∼2 fg PrP^Res^. The relative concentrations of prion seeding activity, i.e., the number of seeding doses giving 50% positive replicate reactions (SD_50_) per unit of tissue, determined by end-point dilution RT-QuIC [41] were 6.42+/−0.38 log SD_50_ per mg brain for 22L and ME7 WT, and 7.92+/−0.63 for RML WT (Figure 2B, light purple bars). These results show that abundant RT-QuIC seeding activity is generated in brain tissue by multiple murine scrapie strains.

### RT-QuIC analysis of anchorless PrP (GPI^−^) brain tissue

**Figure 3:**
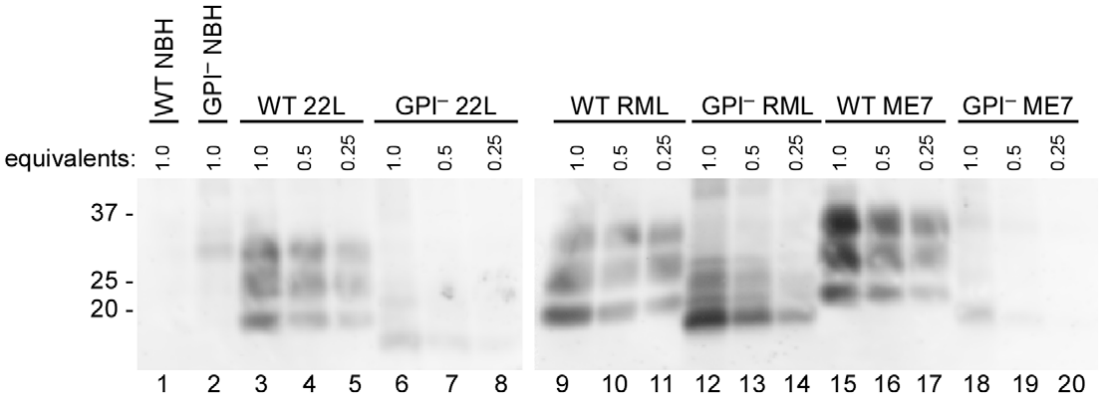
PrP^Res^ levels in brains of GPI^−^ and WT mice infected with multiple mouse-adapted scrapie strains. Normal brain homogenate as well as 22L, RML and ME7-infected brain homogenates were compared by immunoblotting. The sample brain equivalents were loaded into each lane. Lanes 1–2: WT and GPI^−^ NBH undiluted, respectively; Lanes 3–8: WT and GPI^−^ 22L BH undiluted and serially diluted 2-fold and 4-fold; Lanes 9–14: WT and GPI^−^ RML BH undiluted and serially diluted 2-fold and 4-fold; Lanes 15–20: WT and GPI^−^ ME7 BH undiluted and serially diluted 2-fold and 4-fold. A final concentration of 20 µg/mL PK was used to digest brain homogenates. Bands were detected with monoclonal antibody 6D11 as described in materials and methods.

**Figure 4:**
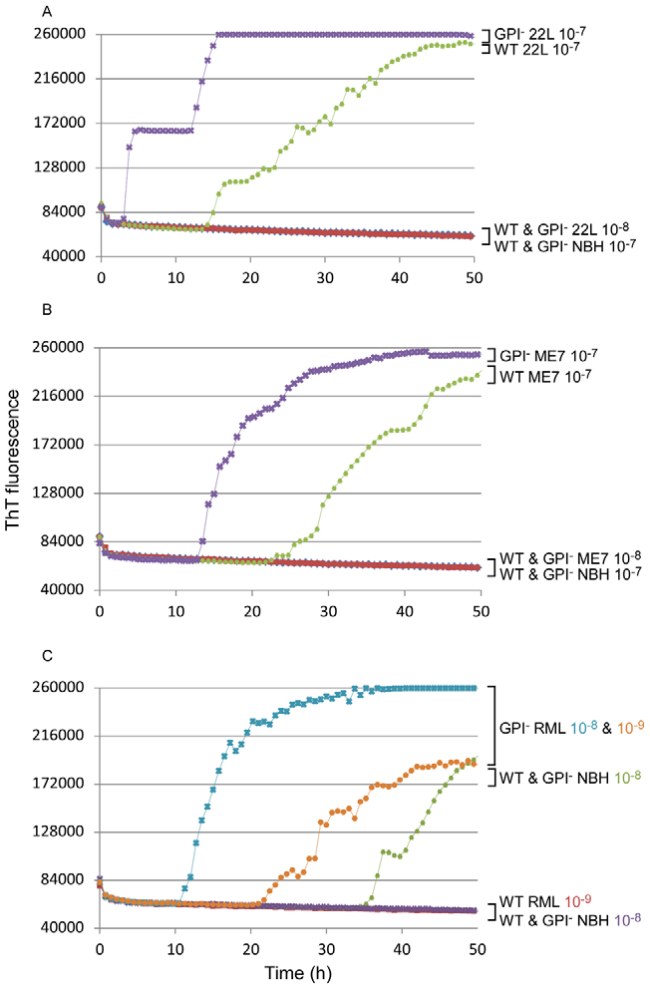
Seeding activity and Log SD_50_ in GPI^−^ and WT mice infected with multiple scrapie strains. RT-QuIC reactions were seeded with 5×10^−7^ and 5×10^−8^ brain dilution from WT and GPI^−^ mice infected with 22L (**A**) and ME7 (**B**) strains; 5×10^−8^ and 5×10^−9^ brain dilutions from WT and GPI^−^ mice infected with RML were compared in (**C**). moPrP^Sen^ 23–231 was used as substrate in all reactions.

**Figure 5:**
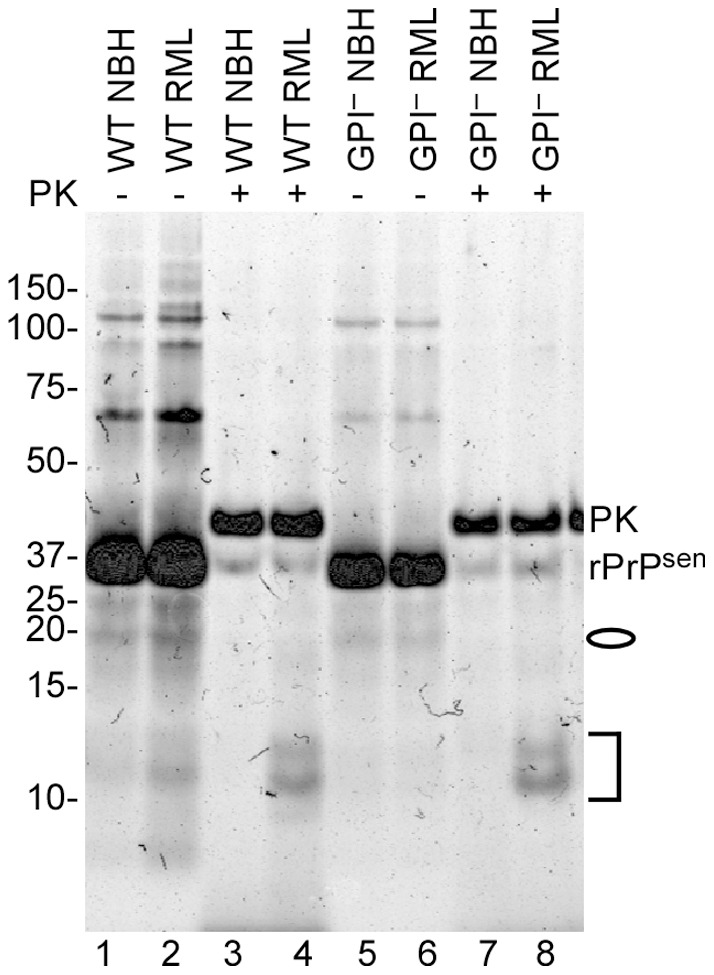
Total protein staining of seeded conversion products from GPI^−^ and WT mice inoculated with RML or normal (NBH) BH. 5×10^−6^ dilutions were used to seed RT-QuIC reactions containing moPrP^C^23–231 substrate. Reaction products were PK digested (+) at final concentration of 10 µg/mL, or not (−) and analyzed by SDS-PAGE. The gel was stained with a total protein stain (Deep Purple). Lanes 1,3: no PK and PK-treated WT uninfected products; Lanes 2,4: no PK and PK-treated WT RML infected products. Lane 5,7: no PK and PK-treated GPI^−^ uninfected products. Lane 6,8: no PK and PK-treated GPI^−^ RML infected products. The oval indicates the weak ∼18 kDa bands while the bracket represents the 12, 13 and 14 kDa bands in the PK-digested products of the scrapie-seeded reactions (lanes 4 and 8).

To evaluate the seeding activity associated with predominantly amyloid forms of PrP^Sc^, we analyzed the same scrapie strains in transgenic mice that express only GPI-anchorless PrP (GPI^−^ mice) [29], [30]. As mentioned above, these mice accumulate PrP^Sc^ that, in contrast to the largely non-amyloid diffuse and amorphous accumulations in wild-type mice, appears to be exclusively contained in amyloid fibrils and plaques. By immunoblotting of PK-treated brain homogenates, the levels of PrP^Res^ present in the brains of the GPI^−^ mice that we tested appeared to be less than or comparable to the levels accumulating in WT mice (Figure 3). However, quantitative immunoblot comparisons of heavily glycosylated, GPI-anchored WT PrP^Res^ with largely unglycosylated, anchorless PrP^Res^ can be difficult due to apparent differences in the binding efficiency and/or immune detection of these types of molecules on blotting membranes [30], [48], [49] (data not shown). Furthermore, PrP^Res^ levels in individual brains can vary markedly during the prolonged and subtle clinical phase of disease in the hemizygous GPI^−^ mice used in this study. Further complicating matters, a recent study reported that analyses by capture ELISA indicated that GPI^−^ mice can accumulate up to 25–50 fold more PrP^Res^ than wild-type mice when inoculated with the RML or ME7 strains of scrapie [50], a conclusion that has differed markedly from at least some immunoblot-based determinations. In any case, our measurements of seeding activity by end-point dilution RT-QuIC [41] using the moPrP^Sen^ 23–231 substrate revealed that hemizygous GPI^−^ mice infected with each scrapie strain had SD_50_ concentrations that were indistinguishable from their WT counterparts (Figure 2B, dark purple bars). Interestingly, the same dilutions of brain homogenates from the GPI^−^ mice gave much shorter lag phases than those from WT mice (Figures 4A–C). Despite these differences in reaction kinetics, we could not detect any difference between the GPI^−^ and WT-seeded (RML) RT-QuIC products with respect to PK-resistant fragments on SDS-PAGE (Figure 5, lanes 4 & 8). Overall, these data indicate that predominantly amyloid forms of PrP^Sc^ have abundant seeding activity and that samples of a given scrapie strain with similar end-point dilutions (i.e. SD_50_/ml) can seed strikingly different RT-QuIC reaction kinetics (i.e. lag phases) depending on whether the host mouse expresses wild-type or GPI^−^ PrP^C^.

### Seeding activity in mice with little PrP^Res^

**Figure 6:**
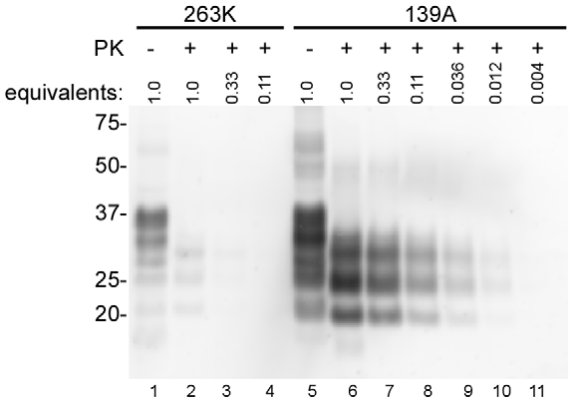
PrP^Res^ levels in synaptosomal fractions from 263K- and 139A-infected 101LL mice by immunoblotting. Lane 1: no PK 101L 263K sample. Lanes 2–4: PK-treated 101L 263K samples undiluted and serially diluted 3-fold and 9-fold. Lane 5: no PK 101L 139A sample. Lanes 6–11: PK-treated 101L139A samples undiluted and serially diluted 3-fold, 9-fold, 27-fold, 81-fold and 243-fold. A final concentration of 100 µg/mL PK was used to digest synaptosomal fractions as described in Materials and Methods. Samples were serially diluted in sample buffer. Bands were detected with monoclonal antibody 6D11.

**Figure 7:**
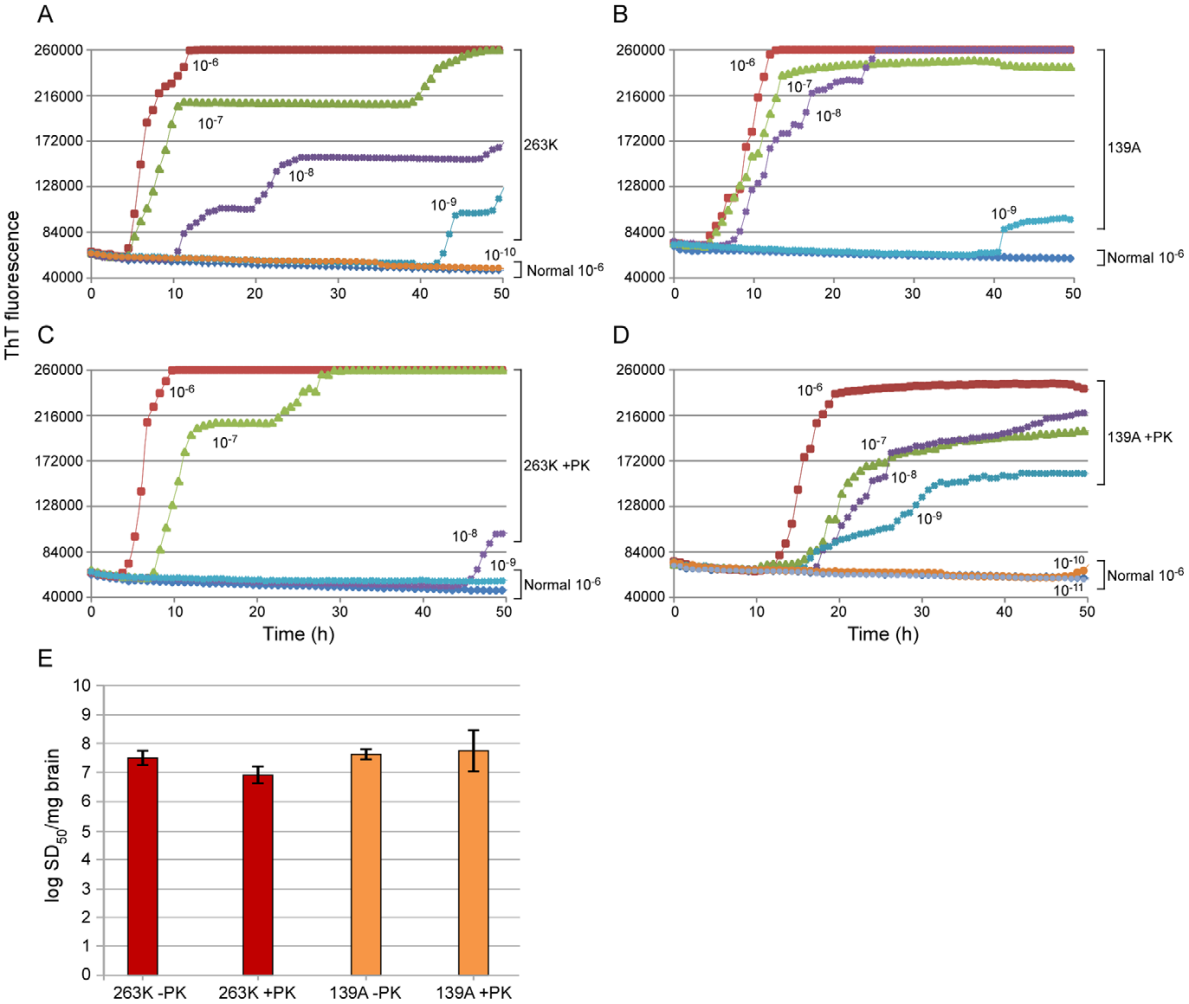
PK-sensitivity of seeding activity in synaptosomes from 101LL mice infected with 263K and 139A. 5×10^−6^ (10^−6^) to 5×10^−11^ (10^−11^) dilutions of PK-treated (100 µg/mL) (**C, D**) or control (-PK) (**A, B**) detergent permeabilized synaptosomal fractions. 263K (**A, C**) and 139A (**B, D**) synaptosomal fractions were seeded into quadruplicate reactions. (**E**) End-point dilution RT-QuIC analysis of 263K (red bars) and 139A (orange bars) strains. The mean ±SD of Spearman-Kärber estimates of the SD_50_/mg brain tissue from three different experiments are shown.

**Figure 8:**
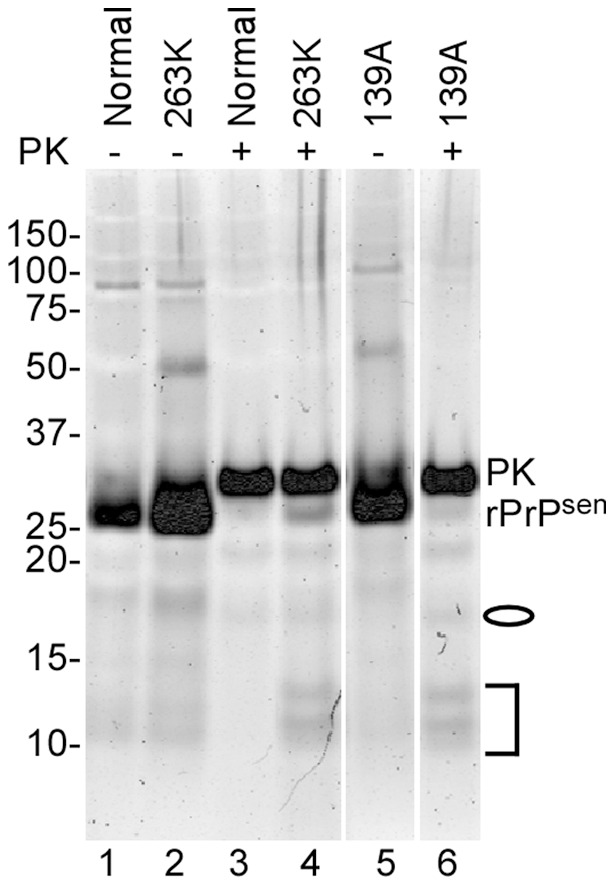
Total protein staining of RT-QuIC conversion products from reactions seeded with synaptosomes from 263K- and 139A-infected 101LL mice. RT-QuIC reactions were seeded with 5×10^−6^ dilutions of synaptosomal fractions. Products were PK digested (+) or not (−) at final concentration of 10 µg/mL and analyzed by SDS-PAGE. The gel was stained with Deep Purple protein stain. Lanes 1,3: no PK and PK treated uninfected products; Lanes 2,4: no PK and PK-treated 263K products. Lanes 5,6: no PK and PK-treated 139A products.The oval indicates the 18 and 19 kDa bands while the bracket represents the 12, 13 and 14 kDa bands in the PK-digested products of the scrapie-seeded reaction.

To determine if prion seeding activity can be detected in hosts with clinical TSE disease but little or no detectable PrP^Res^, we compared two scrapie strains in knock-in transgenic mice homozygous for P101L PrP^C^ (101LL mice) [51]. Inoculation of the 263K scrapie strain causes TSE disease and high infectivity titers in the brain but little or no PrP^Res^ in these mice as detected by immunoblotting and other assays [35]. In contrast, when these animals are inoculated with the 139A scrapie strain, they accumulate readily detectable amounts of PrP^Res^ [37]. Indeed, our immunoblot-based comparisons indicated that brain synaptosome preparations from the 263K-inoculated mice contained ∼81-fold less PrP^Res^ than those from 139A-inoculated mice in the clinical phase of disease (Figure 6). Previous work has shown that a majority of the infectivity fractionates with synaptosomes, and that similar titers are found with these two strains ([35]; unpublished data). Despite the large difference in PrPRes levels with these strains, we measured similar levels of seeding activity by end-point dilution RT-QuIC (Figure 7 A, B, E). Moreover, the profile of PK-resistant bands in the RT-QuIC reaction products was also similar between the two strains (Figure 8, lanes 4 & 6). Altogether, the data indicated abundant seeding activity associated with both high-PrP^Res^ and very low-PrP^Res^ TSE strains.

Next we tested the PK-resistance of the seeding activities associated with the 263K and 139A strains in the 101LL transgenic mice (Figure 7 C, D). Synaptosomes were permeabilized with Triton X-100 and treated with 100 µg/mL PK prior to end-point dilution RT-QuIC. Following PK treatment, little PrP^Res^ was present in the 263K synaptosomes, and the expected size shift in banding pattern was observed in 139A synaptosomes (Figure 6). The PK treatment appeared to cause a modest (∼4-fold) decrease in the mean SD_50_/mg brain value for 263K synaptosomes from three separate experiments (Figure 7E, red bars), but this was of minimal statistical significance (p = 0.056). No effect of PK treatment on the mean SD_50_/mg brain was seen with 139A synaptosomes (Figure 7E, orange bars). Overall, the results suggested that the 263K seeding activity may be somewhat sensitive to PK digestion, but less so than the total synaptosomal PrP content.

### eQuIC detection of prion seeding activity in mouse plasma

**Figure 9:**
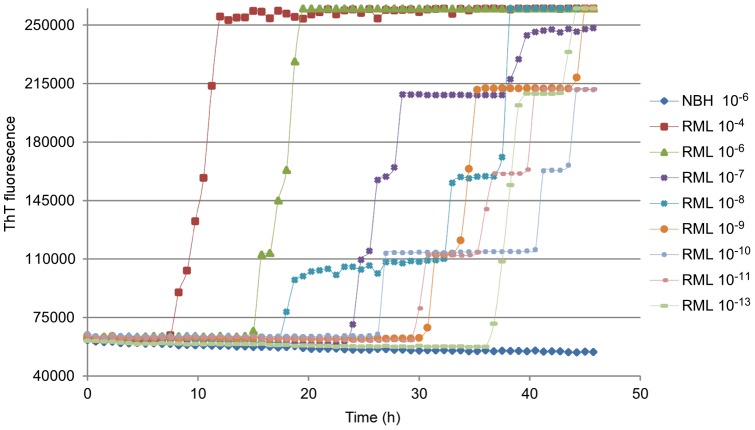
eQuIC detection of RML PrP^Sc^ spiked into mouse plasma without substrate replacement. A 5×10^−6^ dilution of NBH or 5×10^−4^ to 5×10^−13^ dilutions of RML infected brain tissue containing from ∼20 pg to 2 ag of PrP^Res^, respectively, were spiked into 0.2 mL of mouse plasma. PrP^Sc^ was immunoprecipitated using 15B3-coated beads and a portion of the beads was used to seed quadruplicate eQuIC reactions. moPrP^Sen^ 90–231 was used as a substrate in all reactions. The mean ThT fluorescence of the four replicates is shown.

Because blood plasma contains strong inhibitors of RT-QuIC reactions, we used the eQuIC [44] assay to analyze plasma samples from scrapie-infected mice. For this assay, beads coupled with antibody 15B3 [52] were used to capture prion seeding activity from plasma prior to detection by RT-QuIC [44]. Unexpectedly, in contrast to previous results obtained with RT-QuIC alone, the use of moPrP^Sen^ 23–231 substrate with antibody coated beads in the reaction didn't support efficient PrP^Res^ detection. More optimal reaction conditions were observed using moPrP^Sen^ 90–231 as substrate, 300 mM NaCl and 48°C (data not shown). In contrast to the use of this substrate in RT-QuIC as described above, we saw only rare spontaneous ThT-positive responses in negative control reactions under these conditions with beads present in the reaction well (see below). We tested the reaction sensitivity by spiking uninfected mouse plasma with dilutions of brain homogenates from RML-infected mice (Figure 9). We observed positive reactions with dilutions as extreme as 5×10^−13^ in 0.2 mL of plasma, which contained ∼2 ag of PrP^Res^. These results showed that capture of mouse PrP^Res^ with 15B3 antibody allowed the detection of highly diluted mouse seeding activity in plasma and enhanced RT-QuIC sensitivity by ∼10^5^.

In an attempt to improve the reaction speed and sensitivity, we also tried adding fresh substrate to the reaction. This step has been helpful in previously described eQuIC assays for hamster and human prions in plasma samples [44]. However, with the murine-adapted eQuIC system [44], we observed only decreased sensitivity following substrate replacement (data not shown). Thus we abandoned the substrate replacement step in subsequent eQuIC assays for murine prions.

**Figure 10:**
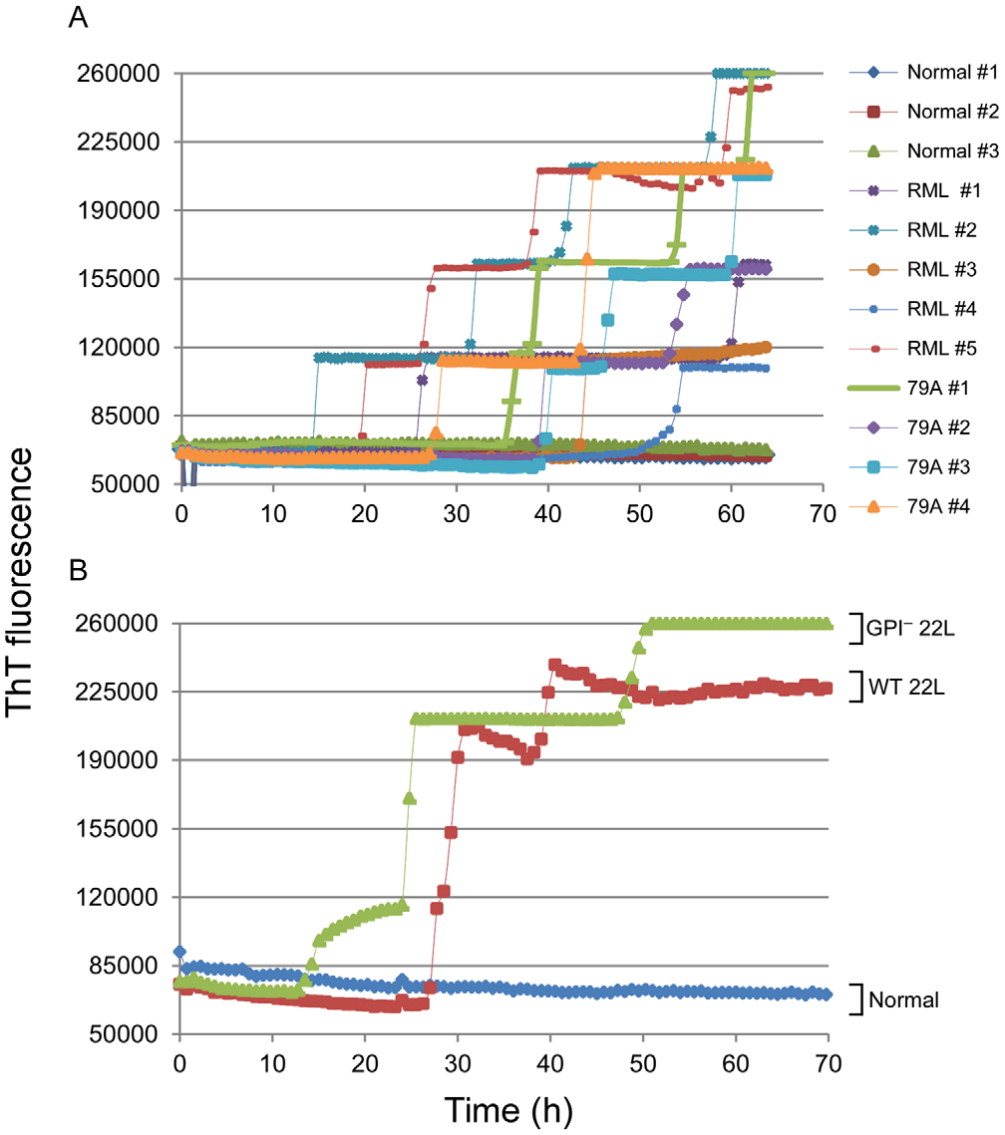
eQuIC detection of endogenous PrP^Sc^ in plasma from clinically ill, scrapie-infected mice. (**A**) plasma samples from five RML-infected, four 79A-infected and three aliquots of plasma pooled from multiple uninfected mice were analyzed. (**B**) Plasma sample analyses from 22L-infected WT and GPI^−^ mice (one each) and pooled plasma from uninfected mice (normal). Endogenous PrP^Sc^ was immunoprecipitated using 15B3-coated beads and a portion of the beads were used to seed quadruplicate eQuIC reactions as described in Materials and Methods.

We also tested whether eQuIC (without substrate replacement) can detect PrP^Sc^ naturally present in the plasma of scrapie-affected mice. Samples were collected in the clinical phase of disease from 9 scrapie-affected WT mice inoculated with, RML or 79A scrapie strains. eQuIC analysis showed that seven of these infected samples gave multiple positive replicate reactions (three with 4/4 positive replicates, two with 3/4 and two with 2/4) while the two remaining scrapie-affected mice gave 1/4 positive replicates (Figure 10A). In contrast, tests of 4 negative control mice gave 0/4 positive replicates, while 1 negative control specimen gave 1/4 positives, with the latter being an apparent false positive occurring late in the reaction (over 55 h). We also got similarly positive reactions (all 4/4 positive replicates) from plasma samples from clinically affected WT and GPI^−^ mice inoculated with 22L scrapie (Figure 10B). Collectively, our data showed the ability of the 15B3-based eQuIC to detect a variety of different mouse-adapted scrapie strains endogenous to plasma in the clinical phase of disease.

## Discussion

Here we demonstrate the *in vitro* amplified detection of mouse-adapted scrapie strains by RT-QuIC and e-QuIC assay. In general, the use of full-length moPrP^Sen^23–231 and low NaCl concentrations allowed rapid and sensitive mouse seed amplification with a very low incidence of false positive reactions in the RT-QuIC. The truncated moPrP^Sen^90–231 substrate tended to undergo spontaneous (prion seed-independent) conversion in RT-QuIC reactions, but, curiously, did not show this tendency in eQuIC reactions. We speculate that the presence of antibody coated beads and/or the altered kinetics of the eQuIC might diminish spontaneous nucleation of moPrP^Sen^90–231. By the same token, interactions of moPrP^Sen^23–231 with the beads might have slowed the eQuIC reaction rate relative to that observed in the absence of the beads. In any case, the versatility of RT-QuIC and eQuIC is indicated by the sensitive detection of several mouse-adapted scrapie strains with divergent PrP^Sc^ characteristics.

Another highly sensitive assay, protein misfolding cyclic amplification (PMCA) [53], has been shown to be capable of amplifying detection of mouse prion strains [54]–[60], but with extended overall reaction time for optimal sensitivity. For instance, Murayama and colleagues were able to detect Chandler (RML) PrP^Res^ in 10^−8^ brain dilution after three rounds of amplification taking >120 hours total [57]. In comparison, we have found that RT-QuIC can detect comparable RML brain dilutions in <40 hours.

Our detection of mouse PrP^Sc^ in plasma extends the use of the e-QuIC, which was shown previously to detect prion seeding activity endogenous to hamster plasma or spiked into human plasma [44]. As with the latter studies, our mouse brain homogenate spiking experiments showed that eQuIC was much more sensitive (∼100,000 fold) than RT-QuIC alone, allowing detection up to 10^−13^-fold dilutions of TSE brain homogenate spiked into plasma.

In contrast to previous eQuIC studies with vCJD and hamster-adapted scrapie [44], and other studies with PMCA [61], the substrate replacement step in the eQuIC protocol was not helpful. The reason for this difference is not clear. However, one possibility is that the murine seed particles are more frangible or less adherent to the beads or surface of the well than are the analogous particles of other host species. If so, then removing reaction fluid to refresh the substrate may deplete the seeds and nascent seeded products and compromise, rather than enhance, the reaction rate and sensitivity. Another possibility is that, relative to other substrates such as hamster and human rPrP^Sen^ [44], mouse rPrP^Sen^ may more readily adopt, and remain in, a state that is readily susceptible to seeded conversion to amyloid; in that case, its replacement would not accelerate the RT-QuIC reaction rate.

A key goal in TSE diagnostics is detection of prion seeding activity in blood. Here we found that 82% of plasma samples from mice clinically affected with multiple scrapie strains gave clear positive reactions. However, in the remainder of the mice the plasma seeding activity levels appeared to be near the detection limit. This could be due to naturally low plasma PrP^Sc^ concentrations, or to the presence of eQuIC inhibitors in plasma. Nevertheless, negative control samples gave no spontaneous conversion of the substrate within 55 h. Further work will be needed to determine if additional gains in sensitivity can be made without increasing the occurrence of false positive reactions.

Our comparison of the WT and GPI^−^ PrP seeds revealed a curious discordance between seed concentration and reaction speed. The reason for the markedly shorter lag phases of RT-QuIC reactions seeded with infected brain from GPI^−^ mice is unclear. Previous work has shown that for a given type of prion seed, lag phases tend to be inversely correlated with seed concentration in RT-QuIC reactions [41], [43]. However, end-point dilution QuIC indicated that the seed, or SD_50_, concentrations in the brains of the GPI^−^ and WT mice that we examined were indistinguishable for a given strain. End-point dilution RT-QuIC should measure primarily the concentration, rather than the relative seeding capacity, of individual seed particles. Clearly, however, PrP^Sc^ seed particles can vary widely in size [13], [31] and presumably other characteristics such as seeding activity per particle [13]. For example larger particles, such as plaques or bundles of fibrils, could have many more seeding surfaces than individual fibrils, protofilaments, or small oligomeric seeds. Given that PrP^Sc^ in GPI^−^ PrP transgenic mice accumulates exclusively in the form of large amyloid fibrils and plaques, we suspect that the average seed particle is larger, with more seeding surfaces, than those in WT brain homogenates. This higher per-particle seeding activity could support faster RT-QuIC kinetics for a given overall seed particle concentration. Alternatively, or additionally, the lack of GPI anchors and/or glycans on the GPI^−^ PrP^Sc^ may allow better access of rPrP^Sen^ substrate molecules to seeding sites on PrP^Sc^ particles, thus improving the rate of conversion per unit seed in the reaction.

The use of the 101LL PrP knock-in transgenic mice allowed us to directly compare, in a single host model, the seeding activities associated with scrapie strains giving high versus unusually low brain levels of PrP^Res^ in the clinical phase of disease. Our observation of similar seed concentrations with the two strains provided evidence that RT-QuIC seeding activity correlates more closely with infectivity levels, which were equivalent, than with PrP^Res^ levels. The seeding activity of the low PrP^Res^ 263K strain, appeared to be marginally more sensitive to PK than that of 139A but neither strain of seed was as sensitive to PK as the vast majority of PrP in the infected brain tissue. Previous work has also failed to identify levels of PK-sensitive PrP^Sc^ in this model that could account for the discrepancy between PrP^Res^ and TSE infectivity [35].

Altogether, we have shown that RT-QuIC: 1) allows highly rapid and sensitive detection of murine prion seeds; 2) works with multiple mouse-adapted scrapie strains and types of tissues (e.g. brain, brain fractions, plasma); and 3) detects diverse types of PrP^Sc^ with different ultrastructures and protease sensitivities, with seeding activity correlating more closely with infectivity than with PrP^Res^ levels. Given the extensive use of mouse TSE models to elucidate the underlying biological principles of prion transmission and pathogenesis, we predict that there will be many interesting applications of the RT-QuIC and eQuIC assays for mouse-adapted TSE strains.

## Materials and Methods

### Recombinant prion protein purification

Genes encoding mouse PrP (residues 23 to 231 and 90–231 accession no.M13685) were amplified and ligated into the pET24 and pET41 vector (Novagen), respectively. Hamster-sheep chimeric PrP (Syrian hamster residues 23 to 137 followed by sheep residues 141 to 234 of the R154 Q171 polymorph [accession no. AY907689]) was amplified and ligated into the pET41 vector (EMD Biosciences), and sequences verified. Protein expression and purification were performed as previously described [41]. Purity of rPrP^Sen^ proteins was ∼99% as estimated by SDS-PAGE, immunoblotting, and mass spectrometry (data not shown).

### Brain tissues homogenate preparation

Wild type C57BL/10 (Prnp+/+) mice and transgenic mice (tg44) expressing only anchorless mouse PrP (GPI^−^ mice) were infected with 22L, ME7 and RML (Chandler) scrapie strains and euthanized at clinical stage of disease by deep isoflurane anesthesia. In the case of the GPI^−^ mice, ME7 inoculations were done with homozygous for the transgene (Tg44+/+), while the RML and 22L scrapie inoculations were done in mice hemizygous for the transgene (Tg44+/−). Brain tissues were collected and 10% (w/v) brain homogenates (BH) were prepared as previously described [30]. Unless otherwise indicated, brain tissues were homogenized using glass Dounce homogenizer in nine volumes (10% w/v) of homogenation buffer (1X PBS pH 7.4, 0.5% Triton X100 and 150 mM NaCl) supplemented with Complete Protease Inhibitor w/EDTA (Roche). For the GPI^−^ ME7 sample, brain tissue was homogenized in nine volumes (10% w/v) of 1X PBS, pH 7.4, with 0.1 mM phenylmethanesulfonylfluoride (PMSF), 1 µg/mL aprotin and 0.7 µg/mL pepstatin A protease inhibitors (Sigma). Following a 2 min 2000×g clarification spin, the supernatant was collected, aliquotted and stored at –80C° at for later use. For spiking experiments and RT-QuIC analyses, BHs were thawed and serially diluted in 0.1% SDS in phosphate-buffered saline (PBS) containing 130 mM NaCl and N2 medium supplement (Gibco) as a source of carrier protein.

### Production of synpatosomal preparations from 101LL infected mice

Brain tissue was harvested from 101LL mice infected with 139A or hamster 263K scrapie following cull by cervical dislocation at a pre-defined clinical endpoint. Brain tissue from animals with confirmed clinical and pathological disease was homogenized in 0.32 M sucrose at 100 mg/mL (w/v) and clarified by centrifugation at 2000×g for 10 min at 4°C. Supernatants were transferred to clean centrifuge tubes, and centrifuged at 12,000×g for 15 min at 4°C. Pellets were washed twice in 0.32 M sucrose before being resuspended in 0.32 M sucrose at 100 mg/mL wet weight tissue equivalent.

### Plasma sample préparation

For plasma collections normal and clinical mice were anesthetized with isoflurane and exsanguinated via heart stick. Blood was immediately transferred to a BD Vacutainer (sodium citrate; Becton-Dickinson) tube and mixed gently. Samples were centrifuged at 3000 rpm in a Eppendorf 5415R centrifuge for 15 min. The plasma fraction was transferred to a new tube and stored at −20°C.

### RT-QuIC

RT-QuIC was performed as previously described [41] except for a few modifications. Briefly, 98 µL of fresh RT-QuIC buffer (10 mM phosphate buffer pH 7.4; 130–400 mM NaCl; 0.1 mg/mL rPrP^Sen^; 10 µM Thioflavin T and 10 µM EDTA) were loaded into wells of a black 96-well plate with a clear bottom (Nunc). Reactions were seeded with 2 µL of the BH or synaptosomal fraction dilutions in a final volume of 100 µL (1∶50 dilution). All reactions contained 0.002% final concentration of SDS. Plates were sealed (Nalgene Nunc International sealer) and incubated in a BMG Fluostar plate reader at 42°C for the designated period with cycles of 1 min shaking (700 rpm double orbital) and 1 min rest throughout the incubation. ThT fluorescence measurements (450+/−10 nm excitation and 480+/−10 nm emission; bottom read) were taken every 45 minutes.

### SD_50_ calculations

SD_50_'s were determined by end point dilution RT-QuIC. In brief, for Spearman-Kärber analysis [62] a dilution series with at least one dilution giving 100% ThT positive replicates and at least one dilution giving 0% ThT positive replicates was chosen. The dilution giving 50% positive replicates was calculated as described [41].

### RT-QuIC products analysis

At the end of the reaction seeded conversion products were recovered from the wells with 0.5% sulphobetaine, treated with 10 µg/mL of PK for 60 min at 37°C, and analyzed by SDS-PAGE. The gel was stained with a total protein stain (Deep Purple, GE Healthcare).

### eQuIC: 15B3 coating of magnetic beads

Rat anti-mouse IgM Dynabeads (Invitrogen) were briefly vortexed and 250 µL of beads (1×10^8^ total beads) were transferred to new tubes for coating. Following incubation on a magnet, bead storage buffer was discarded and the beads washed twice with 5 original suspended bead volumes of coating buffer (0.1% bovine serum albumin in PBS). A final concentration of 0.38 mg/mL of 15B3 antibody (Prionics AG) was used to coat beads in 1 mL of coating buffer.Tubes were incubated with “end-over-end” rotation at room temperature for 2 h. Following three more washes with coating buffer the beads were resuspended in 250 µL coating buffer and stored at 4°C.

### eQuIC of plasma samples

e-QuIC was performed as previously described [44], except for a few modifications. Frozen plasma samples were thawed at 37°C and centrifuged at 16000×g for 1 min. The supernatant was used for 15B3 immunoprecipitation. Pooled normal mouse plasma (Innovative Research) was used as a scrapie-negative control in all experiment. For spiking experiments, centrifuged pooled normal plasma was combined with dilutions of brain homogenates (the latter totaling ≤4% of the plasma volume) before 15B3 immunoprecipitation step. Forty µL of 15B3 coated beads were used per 0.2 mL of plasma. 15B3-coated beads were first captured from the coating buffer with a magnet, the coating buffer was discarded, and 0.2 mL of Immunoprecipitation buffer (IP, Prionics AG) was added. An equal volume of plasma was added and tubes were incubated with “end-over-end” rotation for 24 h at 37°C. The beads were incubated on the magnet for 2 minutes and plasma-IP buffer mixture was discarded. Beads were washed twice with 500 µL of Wash Buffer (WB, Prionics AG) and beads were resuspended in 10 µL of 1XPBS (pH 7.4). The beads were then combined with 0.05% SDS in PBS (1∶1 v/v ratio) and, following incubation at room temperature for 20 min, 5 µL of beads (1∶20 dilution in the plate) were added to 95 µL of eQuIC reaction buffer (10 mM PBS pH 7.4, 300 mM NaCl, 0.1 mg/mL rPrP^sen^, 100 µM ThT, and 10 µM EDTA) in a black 96-well plate with a clear bottom (Nunc).The reaction was incubated in a BMG Fluostar plate reader at 48°C using the same cycles of shake and rest previously described for the RT-QuIC [41].

### Western blotting analysis

PrP^Res^ was detected by immunoblotting. In brief, 10% brain homogenates were digested with 20 µg/mL of proteinase K for 1 h at 37C°. For synaptosome analyses, the fractions were pre-treated with 0.4% Triton X100 (final concentration) and digested with 100 µg/mL of PK with the same conditions as previous described for brain homogenates. PK digestion was stopped with Pefabloc (Roche) at a final concentration of 4 mM. The digested samples were boiled in sample buffer (4 M urea, 4% SDS, 2% β-mercaptoethanol, 8% glycerol, 0.02% bromophenol blue and 50 mM Tris-HCl; pH 6.8) and subjected to SDS-PAGE using 10% BisTris NuPAGE gels (Invitrogen). Proteins were transferred to an Immobilon P membrane (Millipore) using iBlot Gel Transfer System (Life Technologies).The membrane was probed with 6D11 antibody (Covance) at a 1∶10,000 dilution, followed by secondary AP-conjugated antibody goat anti-mouse (1∶10,000 dilution) (Jackson Immuno Research Laboratories). The bands were visualized using the Attophos AP Fluorescent Substrate system (Promega) according to the manufacturer's recommendations.

### Ethics statement

Rocky Mountain Laboratories is an Association for Assessment and Accreditation of Laboratory InternationalCare (AAALAC)-accredited facility, and all animal procedures were carried out in strict accordance with the recommendations in the Guide for the Care and Use of Laboratory Animals of the National Institutes of Health. The protocol was approved by the institution's Animal Use and Care Committee and the National Institutes of Health (Protocol Number: 2010–30). All animal procedures carried out at The Roslin Institute (UK) were approved by the Local Ethical Review Committee, and performed under licence from the UK Home Office, in accordance with the Animals (Scientific Procedures) Act 1986.

## References

[1] Prusiner SB (1998) Prions. Proc Natl Acad Sci U S A 95: 13363–13383. 9811807 10.1073/pnas.95.23.13363 PMC33918

[2] Caughey B, Baron GS, Chesebro B, Jeffrey M (2009) Getting a grip on prions: oligomers, amyloids, anchors and pathological membrane interactions. Annu Rev Biochem 78: 177–204. 19231987 10.1146/annurev.biochem.78.082907.145410 PMC2794486

[3] McKinley MP, Bolton DC, Prusiner SB (1983) A protease-resistant protein is a structural component of the scrapie prion. Cell 35: 57–62. 6414721 10.1016/0092-8674(83)90207-6

[4] Castilla J, Saa P, Hetz C, Soto C (2005) In vitro generation of infectious scrapie prions. Cell 121: 195–206. 15851027 10.1016/j.cell.2005.02.011

[5] Wang F, Wang X, Yuan CG, Ma J (2010) Generating a prion with bacterially expressed recombinant prion protein. Science 327: 1132–1135. 20110469 10.1126/science.1183748 PMC2893558

[6] Kim JI, Cali I, Surewicz K, Kong Q, Raymond GJ, et al (2010) Mammalian prions generated from bacterially expressed prion protein in the absence of any mammalian cofactors. J Biol Chem 285: 14083–14087. 20304915 10.1074/jbc.C110.113464 PMC2863186

[7] Legname G, Baskakov IV, Nguyen HO, Riesner D, Cohen FE, et al (2004) Synthetic mammalian prions. Science 305: 673–676. 15286374 10.1126/science.1100195

[8] Makarava N, Kovacs GG, Bocharova O, Savtchenko R, Alexeeva I, et al (2010) Recombinant prion protein induces a new transmissible prion disease in wild-type animals. Acta Neuropathol 119: 177–187. 20052481 10.1007/s00401-009-0633-x PMC2808531

[9] Kocisko DA, Come JH, Priola SA, Chesebro B, Raymond GJ, et al (1994) Cell-free formation of protease-resistant prion protein. Nature 370: 471–474. 7913989 10.1038/370471a0

[10] Saborio GP, Permanne B, Soto C (2001) Sensitive detection of pathological prion protein by cyclic amplification of protein misfolding. Nature 411: 810–813. 11459061 10.1038/35081095

[11] Prusiner SB, McKinley MP, Bowman KA, Bendheim PE, Bolton DC, et al (1983) Scrapie prions aggregate to form amyloid-like birefringent rods. Cell 35: 349–358. 6418385 10.1016/0092-8674(83)90168-x

[12] Caughey B, Kocisko DA, Raymond GJ, Lansbury PT (1995) Aggregates of scrapie associated prion protein induce the cell-free conversion of protease-sensitive prion protein to the protease-resistant state. Chem & Biol 2: 807–817. 8807814 10.1016/1074-5521(95)90087-x

[13] Silveira JR, Raymond GJ, Hughson AG, Race RE, Sim VL, et al (2005) The most infectious prion protein particles. Nature 437: 257–261. 16148934 10.1038/nature03989 PMC1513539

[14] Diringer H, Gelderblom H, Hilmert H, Ozel M, Edelbluth C, et al (1983) Scrapie infectivity, fibrils and low molecular weight protein. Nature 306: 476–478. 6685822 10.1038/306476a0

[15] Diringer H, Kimberlin RH (1983) Infectious scrapie agent is apparently not as small as recent claims suggest. Biosci Rep 3: 563–568. 12033404 10.1007/BF01120701

[16] Baron GS, Hughson AG, Raymond GJ, Offerdahl DK, Barton KA, et al (2011) Effect of glycans and the glycophosphatidylinositol anchor on strain dependent conformations of scrapie prion protein: improved purifications and infrared spectra. Biochemistry 50: 4479–4490. 21539311 10.1021/bi2003907 PMC3101284

[17] Caughey BW, Dong A, Bhat KS, Ernst D, Hayes SF, et al (1991) Secondary structure analysis of the scrapie-associated protein PrP 27–30 in water by infrared spectroscopy. Biochemistry 30: 7672–7680. 1678278 10.1021/bi00245a003

[18] Smirnovas V, Baron GS, Offerdahl DK, Raymond GJ, Caughey B, et al (2011) Structural organization of brain-derived mammalian prions examined by hydrogen-deuterium exchange. Nat Struct Mol Biol 18: 504–506. 21441913 10.1038/nsmb.2035 PMC3379881

[19] Safar J, Roller PP, Gajdusek DC, Gibbs CJ Jr (1993) Conformational transitions, dissociation, and unfolding of scrapie amyloid (prion) protein. J Biol Chem 268: 20276–20284. 8104185

[20] Pan K-M, Baldwin M, Nguyen J, Gasset M, Serban A, et al (1993) Conversion of alpha-helices into beta-sheets features in the formation of the scrapie prion protein. Proc Natl Acad Sci USA 90: 10962–10966. 7902575 10.1073/pnas.90.23.10962 PMC47901

[21] Field EJ, Raine CS, Joyce G (1967) Scrapie in the rat: an electron-microscope study. I. Amyloid bodies and deposits. Acta Neuropathol 8: 47–56. 4167043 10.1007/BF00686649

[22] Bendheim PE, Barry RA, DeArmond SJ, Stites DP, Prusiner SB (1984) Antibodies to a scrapie prion protein. Nature 310: 418–421. 6431296 10.1038/310418a0

[23] DeArmond SJ, McKinley MP, Barry RA, Braunfeld MB, McColloch JR, et al (1985) Identification of prion amyloid filaments in scrapie-infected brain. Cell 41: 221–235. 3922627 10.1016/0092-8674(85)90076-5

[24] Gadjusek DC (1996) Infectious amyloids: Subacute Spongiform Encephalopathies as Transmissible Cerebral Amyloidoses. In: Fields BN, Knipe DM, Howley PM, editors. Field's Virology. Philadelphia: Lippincott-Raven. 2851–2900.

[25] Bruce ME, McBride PA, Farquhar CF (1989) Precise targeting of the pathology of the sialoglycoprotein, PrP, and vacuolar degeneration in mouse scrapie. Neurosci Lett 102: 1–6. 2550852 10.1016/0304-3940(89)90298-x

[26] Piccardo P, Safar J, Ceroni M, Gajdusek DC, Gibbs CJ Jr (1990) Immunohistochemical localization of prion protein in spongiform encephalopathies and normal brain tissue. Neurology 40: 518–522. 1690364 10.1212/wnl.40.3_part_1.518

[27] Kitamoto T, Tateishi J, Tashima T, Takeshita I, Barry RA, et al (1986) Amyloid plaques in Creutzfeldt-Jakob disease stain with prion protein antibodies. Ann Neurol 20: 204–208. 3092727 10.1002/ana.410200205

[28] Ghetti B, Piccardo P, Spillantini MG, Ichimiya Y, Porro M, et al (1996) Vascular variant of prion protein cerebral amyloidosis with tau-positive neurofibrillary tangles: the phenotype of the stop codon 145 mutation in PRNP. Proc Natl Acad Sci U S A 93: 744–748. 8570627 10.1073/pnas.93.2.744 PMC40125

[29] Chesebro B, Trifilo M, Race R, Meade-White K, Teng C, et al (2005) Anchorless prion protein results in infectious amyloid disease without clinical scrapie. Science 308: 1435–1439. 15933194 10.1126/science.1110837

[30] Chesebro B, Race B, Meade-White K, LaCasse R, Race R, et al (2010) Fatal transmissible amyloid encephalopathy: a new type of prion disease associated with lack of prion protein membrane anchoring. PLoS Pathog 6: e1000800. 20221436 10.1371/journal.ppat.1000800 PMC2832701

[31] Tixador P, Herzog L, Reine F, Jaumain E, Chapuis J, et al (2010) The physical relationship between infectivity and prion protein aggregates is strain-dependent. PLoS Pathog 6: e1000859. 20419156 10.1371/journal.ppat.1000859 PMC2855332

[32] Lasmezas CI, Deslys JP, Robain O, Jaegly A, Beringue V, et al (1997) Transmission of the BSE agent to mice in the absence of detectable abnormal prion protein. Science 275: 402–405. 8994041 10.1126/science.275.5298.402

[33] Safar J, Wille H, Itri V, Groth D, Serban H, et al (1998) Eight prion strains have PrP(Sc) molecules with different conformations [see comments]. Nat Med 4: 1157–1165. 9771749 10.1038/2654

[34] Piccardo P, Manson JC, King D, Ghetti B, Barron RM (2007) Accumulation of prion protein in the brain that is not associated with transmissible disease. Proc Natl Acad Sci U S A 104: 4712–4717. 17360589 10.1073/pnas.0609241104 PMC1838665

[35] Barron RM, Campbell SL, King D, Bellon A, Chapman KE, et al (2007) High titers of transmissible spongiform encephalopathy infectivity associated with extremely low levels of PrPSc in vivo. J Biol Chem 282: 35878–35886. 17923484 10.1074/jbc.M704329200

[36] Sajnani G, Silva CJ, Ramos A, Pastrana MA, Onisko BC, et al (2012) PK-sensitive PrP is infectious and shares basic structural features with PK-resistant PrP. PLoS Pathog 8: e1002547. 22396643 10.1371/journal.ppat.1002547 PMC3291653

[37] Barron RM, Thomson V, King D, Shaw J, Melton DW, et al (2003) Transmission of murine scrapie to P101L transgenic mice. J Gen Virol 84: 3165–3172. 14573822 10.1099/vir.0.19147-0

[38] Barria MA, Gonzalez-Romero D, Soto C (2012) Cyclic amplification of prion protein misfolding. Methods Mol Biol 849: 199–212. 22528092 10.1007/978-1-61779-551-0_14 PMC4068802

[39] Orru CD, Wilham JM, Vascellari S, Hughson AG, Caughey B (2012) New generation QuIC assays for prion seeding activity. Prion 6: 147–152. 22421206 10.4161/pri.19430 PMC7082091

[40] Atarashi R, Satoh K, Sano K, Fuse T, Yamaguchi N, et al (2011) Ultrasensitive human prion detection in cerebrospinal fluid by real-time quaking-induced conversion. Nat Med 17: 175–178. 21278748 10.1038/nm.2294

[41] Wilham JM, Orrú CD, Bessen RA, Atarashi R, Sano K, et al (2010) Rapid End-Point Quantitation of Prion Seeding Activity with Sensitivity Comparable to Bioassays. PLoS Pathog 6: e1001217. 21152012 10.1371/journal.ppat.1001217 PMC2996325

[42] McGuire LI, Peden AH, Orru CD, Wilham JM, Appleford NE, et al (2012) RT-QuIC analysis of cerebrospinal fluid in sporadic Creutzfeldt-Jakob disease. Ann Neurol 72: 278–285. 22926858 10.1002/ana.23589 PMC3458796

[43] Peden AH, McGuire LI, Appleford NE, Mallinson G, Wilham JM, et al (2012) Sensitive and specific detection of sporadic Creutzfeldt-Jakob disease brain prion protein using real-time quaking induced conversion. J Gen Virol 93: 438–449. 22031526 10.1099/vir.0.033365-0 PMC3352348

[44] Orru CD, Wilham JM, Raymond LD, Kuhn F, Schroeder B, et al (2011) Prion disease blood test using immunoprecipitation and improved quaking-induced conversion. MBio 2: e00078–11. 21558432 10.1128/mBio.00078-11 PMC3101782

[45] Orru CD, Hughson AG, Race B, Raymond GJ, Caughey B (2012) Time course of prion seeding activity in cerebrospinal fluid of scrapie-infected hamsters after intratongue and intracerebral inoculations. J Clin Microbiol 50: 1464–1466. 22238438 10.1128/JCM.06099-11 PMC3318555

[46] Bessen RA, Shearin H, Martinka S, Boharski R, Lowe D, et al (2010) Prion Shedding from Olfactory Neurons into Nasal Secretions. PLoS Pathogens 6: e1000837. 20419120 10.1371/journal.ppat.1000837 PMC2855443

[47] Bessen RA, Wilham JM, Lowe D, Watschke CP, Shearin H, et al (2011) Accelerated shedding of prions following damage to the olfactory epithelium. J Virol 86: 1777–1788. 22130543 10.1128/JVI.06626-11 PMC3264367

[48] Nishina KA, Supattapone S (2007) Immunodetection of glycophosphatidylinositol-anchored proteins following treatment with phospholipase C. Anal Biochem. 363: 318–320. 10.1016/j.ab.2007.01.032 PMC1868555 17321480

[49] Kim JI, Surewicz K, Gambetti P, Surewicz WK (2009) The role of glycophosphatidylinositol anchor in the amplification of the scrapie isoform of prion protein in vitro. FEBS Lett 583: 3671–3675. 19854187 10.1016/j.febslet.2009.10.049 PMC2856614

[50] Mahal SP, Jablonski J, Suponitsky-Kroyter I, Oelschlegel AM, Herva ME, et al (2012) Propagation of RML prions in mice expressing PrP devoid of GPI anchor leads to formation of a novel, stable prion strain. PLoS Pathog 8: e1002746. 22685404 10.1371/journal.ppat.1002746 PMC3369955

[51] Manson JC, Jamieson E, Baybutt H, Tuzi NL, Barron R, et al (1999) A single amino acid alteration (101L) introduced into murine PrP dramatically alters incubation time of transmissible spongiform encephalopathy. EMBO J 18: 6855–6864. 10581259 10.1093/emboj/18.23.6855 PMC1171748

[52] Korth C, Stierli B, Streit P, Moser M, Schaller O, et al (1997) Prion (PrPSc)-specific epitope defined by a monoclonal antibody. Nature 390: 74–77. 9363892 10.1038/36337

[53] Saa P, Castilla J, Soto C (2006) Ultra-efficient replication of infectious prions by automated protein misfolding cyclic amplification. J Biol Chem 281: 35245–35252. 16982620 10.1074/jbc.M603964200

[54] Soto C, Anderes L, Suardi S, Cardone F, Castilla J, et al (2005) Pre-symptomatic detection of prions by cyclic amplification of protein misfolding. FEBS Lett 579: 638–642. 15670821 10.1016/j.febslet.2004.12.035

[55] Nishina KA, Deleault NR, Mahal SP, Baskakov I, Luhrs T, et al (2006) The stoichiometry of host PrPC glycoforms modulates the efficiency of PrPSc formation in vitro. Biochemistry 45: 14129–14139. 17115708 10.1021/bi061526k

[56] Castilla J, Gonzalez-Romero D, Saa P, Morales R, de CJ, et al (2008) Crossing the species barrier by PrP(Sc) replication in vitro generates unique infectious prions. Cell 134: 757–768. 18775309 10.1016/j.cell.2008.07.030 PMC2740631

[57] Murayama Y, Yoshioka M, Yokoyama T, Iwamaru Y, Imamura M, et al (2007) Efficient in vitro amplification of a mouse-adapted scrapie prion protein. Neurosci Lett 413: 270–273. 17174030 10.1016/j.neulet.2006.11.056

[58] Green KM, Castilla J, Seward TS, Napier DL, Jewell JE, et al (2008) Accelerated high fidelity prion amplification within and across prion species barriers. PLoS Pathog 4: e1000139. 18769716 10.1371/journal.ppat.1000139 PMC2516356

[59] Tattum MH, Jones S, Pal S, Collinge J, Jackson GS (2010) Discrimination between prion-infected and normal blood samples by protein misfolding cyclic amplification. Transfusion 50: 996–1002. 20180925 10.1111/j.1537-2995.2010.02595.x

[60] Mays CE, Titlow W, Seward T, Telling GC, Ryou C (2009) Enhancement of protein misfolding cyclic amplification by using concentrated cellular prion protein source. Biochem Biophys Res Commun 388: 306–310. 19664595 10.1016/j.bbrc.2009.07.163 PMC2756978

[61] Rubenstein R, Chang B, Gray P, Piltch M, Bulgin MS, et al (2011) Prion disease detection, PMCA kinetics, and IgG in urine from sheep naturally/experimentally infected with scrapie and deer with preclinical/clinical chronic wasting disease. J Virol 85: 9031–9038. 21715495 10.1128/JVI.05111-11 PMC3165845

[62] Dougherty RM (1964) Animal virus titration techniques. In: Harris RJC, editors. Techniques in experimental virology. New York: Academic Press, Inc. 183–186.

